# A New Truncated Lindley-Generated Family of Distributions: Properties, Regression Analysis, and Applications

**DOI:** 10.3390/e25091359

**Published:** 2023-09-20

**Authors:** Mohamed Hussein, Gabriela M. Rodrigues, Edwin M. M. Ortega, Roberto Vila, Howaida Elsayed

**Affiliations:** 1 Department of Mathematics and Computer Science, Alexandria University, Alexandria 21544, Egypt; m.hussein@alexu.edu.eg; 2 Department of Business Administration, College of Business, King Khalid University, Abha 61421, Saudi Arabia; 3 Department of Exact Sciences, University of São Paulo, Piracicaba 13418-900, Brazil; gabrielar@usp.br (G.M.R.); edwin@usp.br (E.M.M.O.); 4 Department of Statistics, University of Brasilia, Brasilia 70910-900, Brazil; rovig161@unb.br

**Keywords:** censored data, survival function, maximum likelihood, regression model, COVID-19 data, 62E10, 62F10, 60E05, 62P10, 62J02

## Abstract

We present the truncated Lindley-*G* (TLG) model, a novel class of probability distributions with an additional shape parameter, by composing a unit distribution called the truncated Lindley distribution with a parent distribution function G(x). The proposed model’s characteristics including critical points, moments, generating function, quantile function, mean deviations, and entropy are discussed. Also, we introduce a regression model based on the truncated Lindley–Weibull distribution considering two systematic components. The model parameters are estimated using the maximum likelihood method. In order to investigate the behavior of the estimators, some simulations are run for various parameter settings, censoring percentages, and sample sizes. Four real datasets are used to demonstrate the new model’s potential.

## 1. Introduction

Suppose that *G* is a cumulative distribution function (cdf) that is defined on the real line, several papers have proposed composing a unit distribution with *G* (a parent cdf) to produce a new cdf. Eugene et al. (2002) [1] combined the cdf of the beta distribution with *G* to create the Beta-*G* model with cdf

$$F\left(x\right)=I_{G(x)}\left(a,b\right),$$

where $I_{x}\left(a,b\right)=\int_{0}^{x}t^{a-1}{\left(1-t\right)}^{b-1}dt/B\left(a,b\right)$ is the regularized incomplete beta function. Alexander et al. (2012) [2] and Nadarajah et al. (2014b) [3] generalized the Beta-*G* to the generalized-Beta-*G* and the modified-Beta-*G*. Cordeiro and Castro (2011) [4] developed the Kumaraswamy-*G* model by combining the Kumaraswamy cdf $F\left(x\right)=1-{\left[{\left(1-x\right)}^{a}\right]}^{b},x\in \left[0,1\right]$ with the parent cdf *G*.

Based on a valid cdf, F(x) for x∈R, for any continuous distribution, we can construct a unit distribution as a truncated version of F(x) with a cdf (monotonically increasing with $\lim_{x\to 0}F\left(x\right)=0$ and $\lim_{x\to 1}F\left(x\right)=1$) given by

(1) $$F_{UT}\left(x\right)=\frac{F(x)}{F(1)},\;\;\;x\in \left[0,1\right],$$

The truncated-*G* (TG) model is constructed by composing this truncated version of the cdf (or its associated survival function $\bar{F}\left(x\right)$) with a parent cdf *G* (or its associated survival function $\bar{G}\left(x\right)$) to give the parent distribution additional modeling ability and produce a new family of univariate distributions with cdfs (monotonically increasing with $\lim_{x\to -\infty }F\left(x\right)=0$ and $\lim_{x\to \infty }F\left(x\right)=1$) given by

(2) $$F_{1}\left(x\right)=F_{UT}\left(G\left(x\right)\right),\;\;\;x\in R,$$

(3) $$F_{2}\left(x\right)=1-F_{UT}\left(1-G\left(x\right)\right),\;\;\;x\in R.$$

A list of TG models are given in Table 1.

In this paper, we generate a new family of continuous distributions using a truncated version of the Lindley distribution.

The new distribution is necessary and helpful because it provides an alternative option for failure time analysis. While there are already numerous existing distributions available for this purpose, having a new distribution adds to the range of choices researchers and analysts have when analyzing failure times. The existing distributions may not always adequately capture the characteristics or behavior of the data being analyzed. Different distributions have different assumptions and properties, and no single distribution can fit all scenarios perfectly. Therefore, having a new distribution can be beneficial in situations where none of the existing options are suitable or provide a good fit to the data. Additionally, the new distribution may offer advantages over existing ones in terms of interpretability, flexibility, or computational efficiency. It could introduce novel features or modeling capabilities that were previously unavailable with other distributions. This can lead to improved accuracy and reliability in failure time analysis.

In summary, while there are already many distributions available for failure time analysis, the introduction of a new distribution expands the options and possibilities for researchers, allowing them to choose the most appropriate model for their specific data and research objectives.

On the other hand, in several research areas (medical, engineering, biology, agronomy, etc.), the failure times are affected by explanatory variables. In this paper, we propose a regression model with censored observations, based on the truncated Lindley–Weibull distribution, which is a feasible alternative for modeling failure time data. Also, different simulation studies are presented to study the behavior of maximum likelihood estimation (MLE), as well as the residual analysis of the proposed regression model. The paper is structured as follows: Section 2 describes the unit truncated Lindley distribution which is the main component of the proposed new model. We discuss its properties, including moments, mode, quantile function (qf), mean deviations, and generating function. Section 3 discusses the proposed TLG model (linear representation, properties, shapes of the TLG, stochastic representation, truncated Lindley–Weibull (TLW) submodel and estimation of the parameters using the maximum likelihood method). In Section 4, we propose a regression model based on the TLW distribution and estimate its parameters using maximum likelihood. Also, we perform some simulation studies for the TLW regression model under different sample sizes and censoring proportions. The TLW regression model application is illustrated by examining four real datasets in Section 5. Finally, Section 6 summarizes the result and presents the conclusions.

## 2. The Unit Truncated Lindley Model

Lindley (1958) [16] first described the Lindley distribution as a lifetime distribution with one parameter. The probability density function (pdf) and the cdf are provided by

$$\begin{array}{ccc} f_{L}\left(x;\theta \right) & = & \frac{\theta^{2}}{\theta +1}\left(1+x\right)e^{-\theta x},\;x>0,\;\theta >0,\;\mathrm{and}\\ F_{L}\left(x;\theta \right) & = & 1-\left(1+\frac{\theta x}{\theta +1}\right)e^{-\theta x},\;x>0,\;\theta >0, \end{array}$$

respectively. We suggest a new unit distribution, the unit truncated Lindley (UTL) distribution, based on the cdf of the Lindley distribution, which is a truncated form of $F_{L}\left(x\right)$ with the cdf and pdf provided by

(4) $$\begin{array}{c} F_{UTL}\left(x\right)=C_{\theta }\left[1+\theta -\left(1+\theta +\theta x\right)\;e^{-\theta \;x}\right]\;x\in \left[0,1\right],\;\theta \neq 0, \end{array}$$

(5) $$\begin{array}{ccc} f_{UTL}\left(x\right) & = & \theta^{2}\;C_{\theta }\left(1+x\right)\;e^{-\theta x}\;x\in \left[0,1\right],\;\theta \neq 0, \end{array}$$

where $C_{\theta }=1/\left(1+\theta -e^{-\theta }-2\theta \;e^{-\theta }\right)>0$.

The properties of the UTL model are given in Appendix A.

## 3. The Truncated Lindley-G Model

The Truncated Lindley-*G* (TLG) model is constructed by applying the TG composition scheme (2) on the cdf of the UTL model given in Equation (4), i.e.,

$$F_{TLG}\left(x\right)=F_{UTL}\left(G\left(x\right)\right).$$

That is, the cdf and pdf of the TLG model are given by

(6) $$\begin{array}{ccc} F_{TLG}\left(x\right) & = & C_{\theta }\;\left[1+\theta -\left(1+\theta +\theta \;G(x)\right)\;e^{-\theta \;G(x)}\right],\;\;\;\;x\in R,\theta \neq 0, \end{array}$$

and

(7) $$\begin{array}{ccc} f_{TLG}\left(x\right) & = & \theta^{2}\;C_{\theta }\;g\left(x\right)\left[1+G(x)\right]\;e^{-\theta G(x)},\;\;\;\;x\in R,\theta \neq 0, \end{array}$$

where $C_{\theta }=1/\left(1+\theta -e^{-\theta }-2\theta \;e^{-\theta }\right)$.

The main reason for choosing the unit truncated form of the Lindley distribution is to add a new parameter to the parent distribution to generate a new distribution. The properties of the generated distribution will need further investigation, as they are, generally, different from those of the parent distribution.

Following the expansion $e^{-\theta G(x)}=\sum_{i=0}^{n}{\left(-1\right)}^{i}{\left[\theta G(x)\right]}^{i}/i!$, the TLG cdf (6) has a linear representation of the exponentiated-*G* (EG) cdf as

(8) $$\begin{array}{c} F_{TLG}\left(x\right)=C_{\theta }\;\left\{1+\theta +\;\sum_{i=0}^{\infty }\nu_{i}\left[\left(\theta +1\right)H_{i}\left(x\right)+\theta H_{i+1}\left(x\right)\right]\right\}. \end{array}$$

where $H_{j}\left(x\right)=G^{j}\left(x\right)$ (for j=i,i+1) is the EG cdf with power parameter *j*.

Differentiating (8) with respect to *x*, we obtain the linear representation of the TLG pdf as follows:

(9) $$\begin{array}{ccc} f_{TLG}\left(x\right) & = & C_{\theta }\;\left\{\;\sum_{i=0}^{\infty }\nu_{i}\left[\left(\theta +1\right)\;h_{i}\left(x\right)+\theta \;h_{i+1}\left(x\right)\right]\right\} \end{array}$$

where $\nu_{i}={\left(-1\right)}^{i+1}\theta^{i}/i!$, $h_{i}\left(x\right)=i\;g\left(x\right)\;G{\left(x\right)}^{i-1}$ and $h_{i+1}\left(x\right)=\left(i+1\right)\;g\left(x\right)\;G{\left(x\right)}^{i}$ are the EG densities with power parameters *i* and i+1, respectively. On the basis of the linear representation (9), some TLG models’ properties are similar to the EG properties reported in several references, such as AL-Hussaini and Ehsanullah (2015) [17]. Henceforth, $Y_{i}$ denotes that an rv has an EG distribution, with power parameter *i* and density $h_{i}\left(x\right)$.

### 3.1. Some Properties of the TLG Model

#### 3.1.1. Critical Points

As $F_{TLG}\left(x\right)=F_{UTL}\left(G\left(x\right)\right)$, we have $f_{TLG}\left(x\right)=g\left(x\right)f_{UTL}\left(G\left(x\right)\right)$. Hence, the derivative of $f_{TLG}\left(x\right)$ is

$$\begin{array}{c} f_{TLG}^{\prime }\left(x\right)=g^{\prime }\left(x\right)f_{UTL}\left(G\left(x\right)\right)+g^{2}\left(x\right)f_{UTL}^{\prime }\left(G\left(x\right)\right). \end{array}$$

Using the identities $f_{UTL}\left(y\right)=\theta^{2}C_{\theta }\left(1+y\right)e^{-\theta y}$ and $f_{UTL}^{\prime }\left(y\right)=\theta^{2}C_{\theta }\left[1-\theta \left(1+y\right)\right]e^{-\theta y}$, the above identity is written as

$$\begin{array}{c} f_{TLG}^{\prime }\left(x\right)=\theta^{2}C_{\theta }e^{-\theta G(x)}\left\{g^{\prime }\left(x\right)\left(1+G\left(x\right)\right)+g^{2}\left(x\right)\left[1-\theta \left(1+G\left(x\right)\right)\right]\right\}. \end{array}$$

Then, all critical points $x_{0}$ of $f_{TLG}$ satisfy $f_{TLG}^{\prime }\left(x_{0}\right)=0$, or equivalently,

(10) $$\begin{array}{c} \left[g^{\prime }\left(x_{0}\right)-\theta g^{2}\left(x_{0}\right)\right]\left(1+G\left(x_{0}\right)\right)+g^{2}\left(x_{0}\right)=0. \end{array}$$

Depending on the choice of the cdf *G*, the above equation can be reduced and its maximum (modes) and minimum points characterized. For an example where the function *G* is chosen to be the Weibull distribution, see Section 3.2.

#### 3.1.2. Moments

Moments allow the examination of some of the distribution’s most significant features and characteristics. The *k*th raw moment (for r=1,2,…) of the TLG model is

$$\begin{array}{ccc} \mu_{k}^{\prime } & = & \int_{-\infty }^{\infty }x^{k}\;f_{TLG}\left(x\right)\;dx=\theta^{2}\;C_{\theta }\int_{-\infty }^{\infty }x^{k}g\left(x\right)\left[1+G(x)\right]\;e^{-\theta G(x)}dx\\ & = & \theta^{2}\;C_{\theta }\int_{0}^{1}{\left[Q_{G}\left(y\right)\right]}^{k}\left[1+y\right]\;e^{-\theta y}dy, \end{array}$$

where $Q_{G}$ is the qf associated with the parent cdf *G*.

Furthermore, the *k*th raw moment can be expressed from (9) using the moments of the EG distribution as

$$\mu_{r}^{\prime }=C_{\theta }\;\left\{\;\sum_{i=0}^{\infty }\nu_{i}\left[\left(\theta +1\right)\;E\left(Y_{i}^{r}\right)+\theta \;E\left(Y_{i+1}^{r}\right)\right]\right\}.$$

#### 3.1.3. Quantile Function

The qf is a highly desirable property in statistical distributions and is especially helpful in the computation of several values in statistical modeling and inferences. By inverting the cdf of the TLG distribution in (6), the qf for the TLG distribution can be expressed using the qf associated with the parent cdf *G* as

(11) $$\begin{array}{ccc} Q_{TLG}\left(u\right) & = & Q_{G}\left\{-1-\frac{1}{\theta }-\frac{1}{\theta }W\left[\left(uC_{\theta }^{-1}-\theta -1\right)e^{-\theta -1}\right]\right\},\;\;\;\;u\in \left(0,1\right) \end{array}$$

Therefore, $X=Q_{G}\left(U\right)$ follows the TLG distribution with pdf (7) if *U* is a uniform variate on the unit interval.

#### 3.1.4. Mean Deviations

The following relationships can be used to describe, respectively, the mean deviations of *X* about the mean μ=E(X) and the median *M*.

$$\begin{array}{ccc} \delta_{1} & = & \int_{-\infty }^{\infty }|x-\mu |\;f_{TLG}\left(x\right)\;dx=2\mu \;F\left(\mu \right)-2C_{\theta }\;\sum_{i=0}^{\infty }\nu_{i}\;\;\left[\left(\theta +1\right)\;I_{i}\left(\mu ,1\right)+\theta \;I_{i+1}\left(\mu ,1\right)\right],\mathrm{and}\\ \delta_{2} & = & \int_{-\infty }^{\infty }|x-M|\;f_{TLG}\left(x\right)\;dx=\mu -2C_{\theta }\;\sum_{i=0}^{\infty }\nu_{i}\;\;\left[\left(\theta +1\right)\;I_{i}\left(M,1\right)+\theta \;I_{i+1}\left(M,1\right)\right], \end{array}$$

where $I_{j}\left(t,k\right)$ is the *k*th incomplete moment of the rv $Y_{j}$ that has an EG distribution with power parameter *j* (i.e., $Y_{j}∼h_{j}\left(x\right)$).

#### 3.1.5. Moment Generating Function

The mgf of X∼TLG can be expressed in an integral form as

$$\begin{array}{ccc} M_{X}\left(t\right) & = & E\left(e^{tX}\right)=\int_{-\infty }^{\infty }\;e^{tx}f_{TLG}\left(x\right)dx\\ & = & \theta^{2}\;C_{\theta }\;\int_{-\infty }^{\infty }\;g\left(x\right)\left[1+G(x)\right]e^{-\left[\theta G(x)-tx\right]}\;dx\\ & = & \theta^{2}\;C_{\theta }\;\int_{0}^{1}\left[1+y\right]e^{-\left[\theta y-t\;Q_{G}\left(y\right)\right]}\;dy. \end{array}$$

Furthermore, it can be expressed using the mgf of the EG distribution as

$$\begin{array}{ccc} M_{X}\left(t\right) & = & C_{\theta }\;\left\{\;\sum_{i=0}^{\infty }\nu_{i}\left[\left(\theta +1\right)\;M_{i}\left(t\right)+\theta \;M_{i+1}\left(t\right)\right]\right\}, \end{array}$$

where $M_{j}\left(t\right)$ is the mgf of an rv $Y_{j}$ that has an EG distribution with power parameter *j* ($Y_{j}∼h_{j}\left(x\right)$).

#### 3.1.6. Entropy

Entropy measures the change in the uncertainty in physical systems. The Shannon and Rényi entropies are two well-known entropy measurements. Entropy values range from very small to very large, with larger values indicating greater data uncertainty. In this section, we derive the continuous Rényi and Shannon entropies of the TLG distribution. The Rényi entropy, R(τ) where τ>0, τ≠1 of the TLG distribution is given by

$$R\left(\tau \right)=\frac{1}{1-\tau }\log\int_{-\infty }^{\infty }f_{TLG}^{\tau }\left(x\right)dx=\frac{1}{1-\tau }\log\left[\theta^{2\tau }\;C_{\theta }^{\tau }\int_{-\infty }^{\infty }g{\left(x\right)}^{\tau }{\left[1+G(x)\right]}^{\tau }\;e^{-r\;\theta \;G(x)}dx\right].$$

It follows from the expansions 1+G(x)r=∑j=0∞rjG(x)j and $e^{-r\;\theta G(x)}=\sum_{i=0}^{n}\frac{{\left(-1\right)}^{i}}{i!}{\left[r\;\theta \;G(x)\right]}^{i}$ that

R(τ)=11−τlogθ2τCθτ∑j=0τ∑i=0nτj(−1)iτθii!∫−∞∞g(x)τG(x)i+jdx.

The Shannon entropy of the TLG distribution is given

$$\begin{array}{c} S\left(\tau \right)=-E\left[\log f_{TLG}\left(X\right)\right]=-\log\left(\theta^{2}\;C_{\theta }\right)-E\left[\log g(X)\right]-E\left[\log(1+G(X))\right]+\theta E\left[G(X)\right], \end{array}$$

using the expansion

$$\begin{array}{c} \log\left[1+G(x)\right]=\sum_{i=1}^{\infty }\frac{{\left(-1\right)}^{i+1}}{i}\;G^{i}\left(x\right), \end{array}$$

we have

$$\begin{array}{c} \eta =-\log\left(\theta^{2}\;C_{\theta }\right)+\eta_{G}-\sum_{i=1}^{\infty }\frac{{\left(-1\right)}^{i+1}}{i}\;E\left[G^{i}\left(X\right)\right]+\theta E\left[G(X)\right], \end{array}$$

where $\eta_{G}$ is the Shannon entropy for the parent distribution. Since G(X)∼U(0,1), then

$$\begin{array}{ccc} \eta & = & -\log\left(\theta^{2}\;C_{\theta }\right)+\eta_{G}-\sum_{i=1}^{\infty }\frac{{\left(-1\right)}^{i+1}}{i(i+1)}+\frac{\theta }{2},\\ & = & -\log\left(\theta^{2}\;C_{\theta }\right)+\eta_{G}+1-2\log2+\frac{\theta }{2}. \end{array}$$

### 3.2. Truncated Lindley–Weibull (TLW) Model

Consider the parent distribution is the Weibull distribution with shape parameter k>0, and scale parameter λ>0, the cdf and pdf are given by

(12) $$\begin{array}{c} \begin{array}{ccc} G(x) & = & G\left(x;k,\lambda \right)=1-e^{-{\left(x/\lambda \right)}^{k}},\mathrm{and}\\ g(x) & = & g\left(x;k,\lambda \right)=\frac{k}{\lambda }{\left(\frac{x}{\lambda }\right)}^{k-1}e^{-{\left(x/\lambda \right)}^{k}},\;\;\;\;x>0. \end{array} \end{array}$$

The cdf and pdf of the truncated Lindley–Weibull (TLW) model are given by

(13) $$F_{TLW}\left(x\right)=C_{\theta }\;\left\{1+\theta -\left[1+\theta +\theta \left(1-e^{-{\left(x/\lambda \right)}^{k}}\right)\right]e^{-\theta \left(1-e^{-{\left(x/\lambda \right)}^{k}}\right)}\right\},\;\;\;x,k,\lambda >0,\theta \neq 0,\mathrm{and}$$

(14) $$\begin{array}{c} f_{TLW}\left(x\right)=\frac{k\;\theta^{2}\;C_{\theta }}{\lambda }{\left(\frac{x}{\lambda }\right)}^{k-1}\left(2-e^{-{\left(x/\lambda \right)}^{k}}\right)e^{-{\left(x/\lambda \right)}^{k}-\theta \left(1-e^{-{\left(x/\lambda \right)}^{k}}\right)},\;\;\;x,\;k,\;\lambda >0,\;\theta \neq 0, \end{array}$$

respectively, where $C_{\theta }$ is as in Equation (7). Note that

(15) $$\begin{array}{c} \lim_{x\to 0^{+}}f_{TLW}\left(x\right)=\left\{\begin{array}{cc} \infty , & k<1,\\ \frac{\theta^{2}C_{\theta }}{\lambda }, & k=1,\\ 0, & k>1, \end{array}\right.\;\mathrm{and}\;\lim_{x\to \infty }f_{TLW}\left(x\right)=0. \end{array}$$

**Figure 1:**
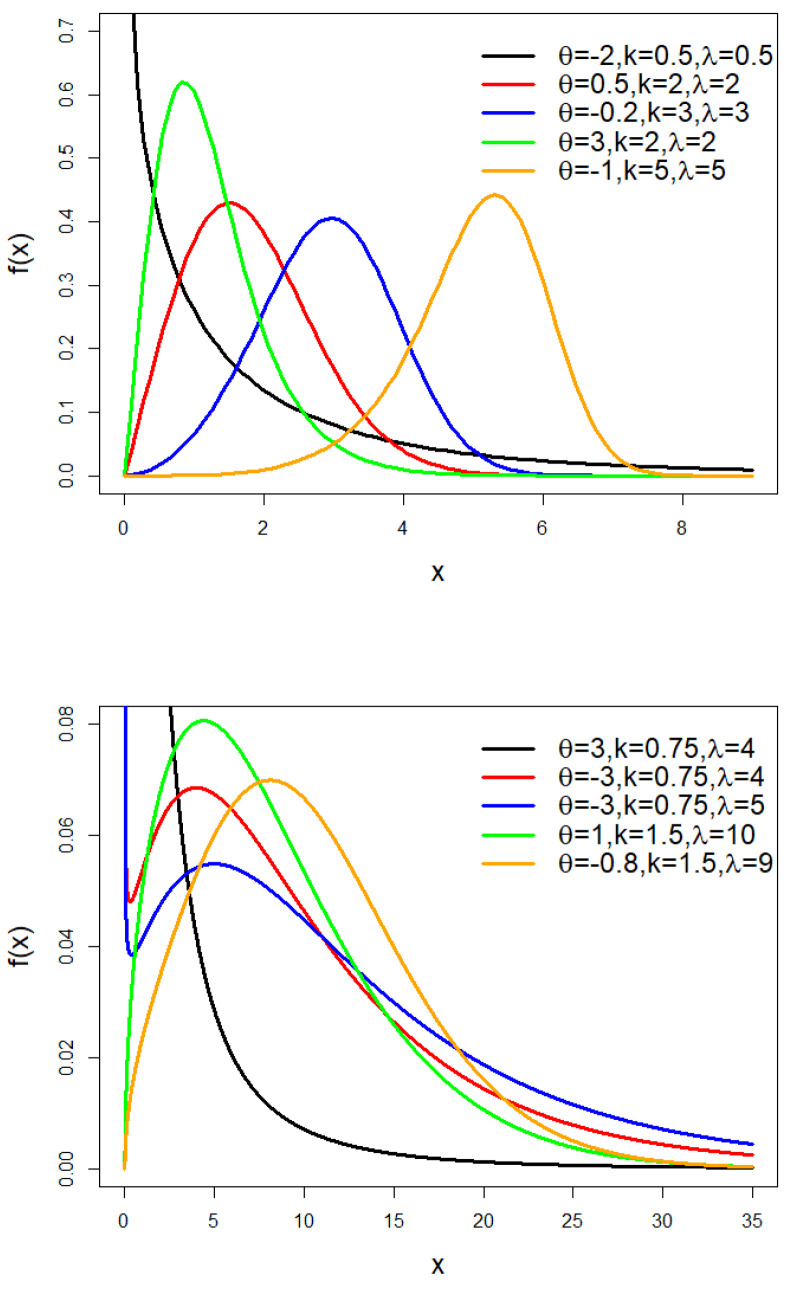
The pdf of the TLW model.

The TLW model’s pdf is shown in Figure 1 for various values of θ,k, and λ. Figure 1 illustrates how the TLW distribution’s density function is flexible and changes in shape depending on the parameter values.

#### 3.2.1. Shapes of the TLW pdf

Considering *G* and *g* as given in (12), the Equation (10) of critical points is written as

$$\begin{array}{c} 0=\left[g^{\prime }\left(x_{0}\right)-\theta g^{2}\left(x_{0}\right)\right]\left(1+G\left(x_{0}\right)\right)+g^{2}\left(x_{0}\right)=\left[g^{\prime }\left(x_{0}\right)-\theta g^{2}\left(x_{0}\right)\right]\left[2-e^{-{\left(x_{0}/\lambda \right)}^{k}}\right]+g^{2}\left(x_{0}\right). \end{array}$$

As $g^{\prime }\left(x\right)=-g\left(x\right)\left\{k[{\left(x/\lambda \right)}^{k}-1]+1\right\}/x$, the above identity becomes

$$\begin{array}{c} 0=-g\left(x_{0}\right)\left\{\frac{k[{\left(x_{0}/\lambda \right)}^{k}-1]+1}{x_{0}}+\theta g\left(x_{0}\right)\right\}\left[2-e^{-{\left(x_{0}/\lambda \right)}^{k}}\right]+g^{2}\left(x_{0}\right). \end{array}$$

Since $g\left(x\right)=\left(k/\lambda \right){\left(x/\lambda \right)}^{k-1}e^{-{\left(x/\lambda \right)}^{k}}$ and $g(x_{0})>0$ for each $x_{0}>0$, the above identity is equivalently written as

(16) $$\begin{array}{c} A\left(z_{0}\right)=B_{\theta ,k}\left(z_{0}\right), \end{array}$$

where for $z_{0}={\left(x_{0}/\lambda \right)}^{k}$ and θ≠0, we denote

$$\begin{array}{c} A\left(z_{0}\right)\equiv -z_{0}e^{-z_{0}},\;B_{\theta ,k}\left(z_{0}\right)\equiv \frac{2}{\theta }\;\frac{z_{0}\left(1-e^{-z_{0}}\right)}{2-e^{-z_{0}}}+\tau^{*}\;\mathrm{and}\;\tau^{*}\equiv \frac{1}{\theta }\left(\frac{1-k}{k}\right). \end{array}$$

A simple calculation shows that the function $z_{0}\mapsto B_{\theta ,k}\left(z_{0}\right)$ is increasing (respectively, decreasing) when θ>0 (respectively, θ<0). Furthermore, notice that the function $z_{0}\mapsto A\left(z_{0}\right)$ reaches the minimum value −1/e at $z_{0}=1$. Using the graphs of the functions *A* and $B_{\theta ,k}$, and varying the parameters θ and $\tau^{*}$, we can find the points of intersection of both graphs. Therefore, we can compactly classify the number of roots of Equation (16), as indicated in Table 2.

Based on Table 2, in what follows we divide our analysis into the following cases.

1 If θ>0
  (a) and $\tau^{*}⩽-1/e$, then k>1 and, by Table 2, there is a single root, $z_{0}={\left(x_{0}/\lambda \right)}^{k}$, of Equation (16). That is, $x_{0}=\lambda z_{0}^{1/k}$, with k>1, is a single critical point of $f_{TLW}$. But, by (15), $\lim_{x\to 0^{+}}f_{TLW}\left(x\right)=\lim_{x\to \infty }f_{TLW}\left(x\right)=0$ for k>1. Consequently, $x_{0}$ is a single maximum point of the TLW pdf. Hence, for θ>0 and $\tau^{*}⩽-1/e$, the TLW pdf is unimodal with mode $x_{0}$.
  (b) and $-1/e<\tau^{*}<0$, then k>1. Following the same steps as in Item 1(a) we have that $f_{TLW}$ is unimodal.
  (c) and $\tau^{*}⩾0$, then k⩽1 and, by Table 2, there is no root of Equation (16). I.e., there is no critical point of $f_{TLW}$. But, by (15), $\lim_{x\to 0^{+}}f_{TLW}\left(x\right)=\infty$ for k<1 (and $=\theta^{2}C_{\theta }/\lambda$ for k=1) and $\lim_{x\to \infty }f_{TLW}\left(x\right)=0$. Consequently, for θ>0 and $\tau^{*}⩾0$, the TLW pdf is decreasing.
2 If θ<0
  (a) and $\tau^{*}⩽-1/e$, then k<1. Following the same steps as in Item 1(c) we have that $f_{TLW}$ is decreasing.
  (b) and $-1/e<\tau^{*}<0$, then k<1, by Table 2, there are two roots, $z_{0}={\left(x_{0}/\lambda \right)}^{k}$ and $z_{1}={\left(x_{1}/\lambda \right)}^{k}$, of Equation (16). In other words, $x_{0}=\lambda z_{0}^{1/k}$ and $x_{1}=\lambda z_{1}^{1/k}$, with k<1, are two critical points of $f_{TLW}$. Without loss of generality, assume that $x_{0}<x_{1}$. By (15), $\lim_{x\to 0^{+}}f_{TLW}\left(x\right)=\infty$, for k<1, and $\lim_{x\to \infty }f_{TLW}\left(x\right)=0$. Consequently, $f_{TLW}$ has an decreasing–increasing–decreasing shape with minimum point $x_{0}$ and maximum point $x_{1}$.
  (c) and $\tau^{*}⩾0$, then k⩽1. Following the same steps as in Item 1(a) we have that $f_{TLW}$ is unimodal.

Table 3 summarizes the shapes of $f_{TLW}$ obtained in Items 1 and 2 above.

Note that the parameters θ and $\tau^{*}$ obtained from Figure 1 obey the pdf shapes obtained in Table 3.

**Figure 2:**
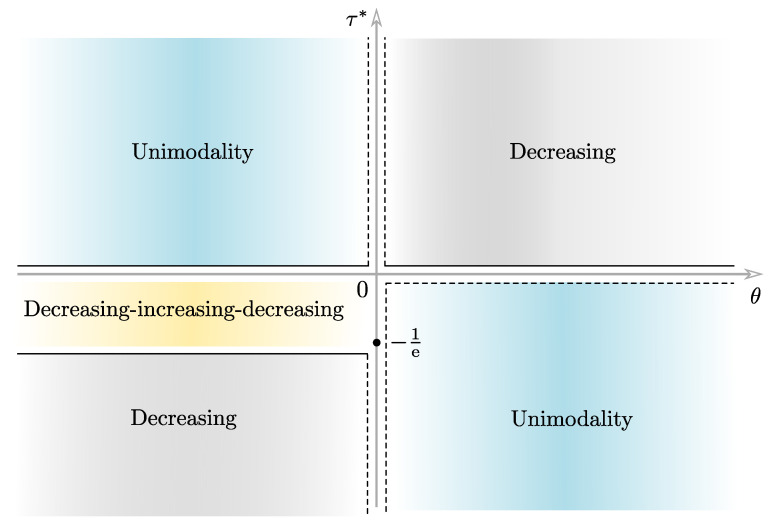
Regions of the Cartesian plane $\theta \tau^{*}$ where different forms of the TLW pdf occur.

By way of illustration in Figure 2, we represent the shapes of the TLW pdf shown in Table 3.

#### 3.2.2. Stochastic Representation

Let *X* and *Y* be two random variables with TLW and UTL distributions, respectively. As $F_{TLW}\left(x\right)=F_{UTL}\left(G\left(x\right)\right)$ with $G\left(x\right)=1-e^{-{\left(x/\lambda \right)}^{k}}$, we obtain

$$\begin{array}{c} F_{TLW}\left(x\right)=P\left(X⩽x\right)=P\left(Y⩽G\left(x\right)\right)=P\left(G^{-1}\left(Y\right)⩽x\right)=P\left(\lambda {\left[-\log\left(1-Y\right)\right]}^{1/k})⩽x\right),\;\forall x. \end{array}$$

Therefore, *X* has the stochastic representation

$$\begin{array}{c} X\overset{d}{=}\lambda {\left[-\log\left(1-Y\right)\right]}^{1/k}, \end{array}$$

with $\overset{d}{=}$ being equality in distribution. In addition to generating random numbers, a stochastic representation is useful for determining moments, characteristic functions, quantiles, etc.

### 3.3. Maximum Likelihood Estimation

Let $x_{1},\cdots ,x_{n}$ represent the observed values from the TLW model with the pdf given in (14). For the vector of parameters $\Theta ={\left(\theta ,k,\lambda \right)}^{⊤}$, the log-likelihood function is provided by

(17) $$\begin{array}{ccc} \ell =\ell (\Theta ) & = & n\left[\log\theta^{2}+\log C_{\theta }+\log k-k\;\log\lambda \right]+\left(k-1\right)\sum_{i=0}^{n}\log x_{i}\\ & & +\sum_{i=0}^{n}\log\left[2-e^{-{\left(x_{i}/\lambda \right)}^{k}}\right]-\theta \sum_{i=0}^{n}\left[1-e^{-{\left(x_{i}/\lambda \right)}^{k}}\right]. \end{array}$$

The following are the elements comprising the score vector U(Θ)

$$\begin{array}{ccc} U_{\theta } & = & n\;\left[\frac{2}{\theta }-\frac{1-e^{-\theta }+2\theta \;e^{-\theta }}{1+\theta -e^{-\theta }-2\theta \;e^{-\theta }}+\frac{1}{k}-\frac{1}{\lambda }\right]-\sum_{i=0}^{n}\left[1-e^{-{\left(x_{i}/\lambda \right)}^{k}}\right],\\ U_{k} & = & \frac{n}{k}-n\log\lambda +\sum_{i=0}^{n}\log x_{i}-\frac{n}{\lambda^{k}}\sum_{i=1}^{n}x_{i}^{k}+\frac{1}{\lambda^{k}}\sum_{i=1}^{n}\frac{x_{i}^{k}\;\left(\log x_{i}-\log\lambda \right)\;e^{-{\left(x_{i}/\lambda \right)}^{k}}}{2-e^{-{\left(x_{i}\lambda \right)}^{k}}}\\ & & -\frac{\theta }{\lambda^{k}}\sum_{i=0}^{n}x_{i}^{k}\left(\log x_{i}-\log\lambda \right)e^{-{\left(x_{i}/\lambda \right)}^{k}},\\ U_{\lambda } & = & -\frac{n\;k}{\lambda }+\frac{k}{\lambda^{k+1}}\sum_{i=0}^{n}x_{i}^{k}+\frac{k}{\lambda^{k+1}}\sum_{i=1}^{n}\frac{x_{i}^{k}\;e^{-{\left(x_{i}/\lambda \right)}^{k}}}{2-e^{-{\left(x_{i}/\lambda \right)}^{k}}}+\frac{k\;\theta }{\lambda^{k+1}}\sum_{i=0}^{n}x_{i}e^{-{\left(x_{i}/\lambda \right)}^{k}}. \end{array}$$

Traditionally, the MLEs of the three parameters can also be calculated by setting the preceding equations to zero and simultaneously solving them. Since it appears impossible to find a closed form estimator for Θ, direct maximization of (17), as a multidimensional nonlinear unconstrained function, via a quasi-Newton optimization technique such as BFGS, SANN, Nelder–Mead, or CG might be appropriate for finding the maximum likelihood estimates of $\Theta ={\left(\theta ,k,\lambda \right)}^{⊤}$.

### 3.4. Monte Carlo Simulation

By generating *n* observations from the TLW distribution with varying parameter values, we conduct simulations to validate the performance of the MLEs of the TLW distribution parameters. The BFGS method from the R package is utilized to estimate the parameter values. The sample sizes considered are *n* = 20, 50, 100, 150, and 300, and the replicates number is *N* = 5000. The simulation results are evaluated using the mean absolute bias (MAB), the mean square error (MSE), and the average estimates (AEs), where for $\Theta ={\left(\theta ,k,\lambda \right)}^{⊤}$ we have

(18) $$MAB\left(\hat{\Theta }\right)=\frac{1}{N}\sum_{i=1}^{N}\left|\hat{\Theta }-\Theta \right|,\;MSE\left(\hat{\Theta }\right)=\frac{1}{N}\sum_{i=1}^{N}{\left(\hat{\Theta }-\Theta \right)}^{2},\;AE\left(\hat{\Theta }\right)=\frac{1}{N}\sum_{i=1}^{N}\hat{\Theta_{i}}.\;$$

The results in Table 4 and Table 5 show that the AEs tend to the true values and that the MABs and MSEs vanish as *n* increases, which reveals the asymptotic consistency of the MLEs of the TLW parameters.

Using Equation (11), for the Weibull distribution we have $Q_{W}\left(u\right)=\lambda \;{\left[-\log\left(1-u\right)\right]}^{\frac{1}{k}}$, implying that the qf of the TLW distribution is

$$Q_{TLW}\left(u\right)=\lambda {\left\{-\log\left[2+\frac{1}{\theta }+\frac{1}{\theta }W_{-1}\left(\left(uC_{\theta }^{-1}-\theta -1\right)\;e^{-\theta -1}\right)\right]\right\}}^{\frac{1}{K}}.$$

The data are generated from

$$X=\lambda {\left\{-\log\left[2+\frac{1}{\theta }+\frac{1}{\theta }W_{-1}\left(\left(U\;C_{\theta }^{-1}-\theta -1\right)\;e^{-\theta -1}\right)\right]\right\}}^{\frac{1}{K}},\;\;U∼U\left(0,1\right).$$

## 4. The TLW Regression Model with Censored Data and Two Systematic Components

Statistical analysis of lifetimes is an important topic used in different areas such as, for example, medicine, biology, epidemiology, engineering, among others. Failure time refers to the time until the occurrence of an event of interest, which may be death, the appearance of a tumor, the development of a disease, the breakdown of an electronic component, among other examples.

We relate the parameters λ and *k* to

$v={\left(v_{1},\ldots ,v_{p}\right)}^{T}$ covariates by the logarithm link function

$$\lambda_{i}=\exp\left(v_{i}^{T}\beta_{1}\right)\;and\;k_{i}=\exp\left(v_{i}^{T}\beta_{2}\right),\;i=1,\ldots ,n,$$

respectively, where $\beta_{1}={\left(\beta_{11},\ldots ,\beta_{1p}\right)}^{T}$ and $\beta_{2}={\left(\beta_{21},\ldots ,\beta_{2p}\right)}^{T}$ denote the vectors of regression coefficients and $v_{i}^{T}=\left(v_{i1},\ldots ,v_{ip}\right)$.

The survival function of X|v is given by

(19) $$\begin{array}{c} S\left(x|v\right)=1-c_{\theta }\left\{1+\theta -\left[1+\theta +\omega (x|v)\right]\exp\left[-\omega \left(x|v\right)\right]\right\}, \end{array}$$

where

$$\omega \left(x|v\right)=\theta \left\{1-\exp\left[-{\left(\frac{x}{\exp(v^{T}\beta_{1})}\right)}^{\exp\left(v^{T}\beta_{2}\right)}\right]\right\}.$$

Equation (19) is referred to as the TLW parametric regression model. This regression model opens new possibilities for fitting many different types of data.

Consider a sample $\left(x_{1},v_{1}\right),\ldots ,\left(x_{n},v_{n}\right)$ of *n* independent observations, where each random response is defined by $x_{i}=\min\left\{x_{i}^{*},c_{i}\right\}$, where $c_{1},\cdots ,c_{n}$ are the censoring times and $x_{1}^{*},\cdots ,x_{n}^{*}$ are the observed lifetimes. We assume non-informative censoring such that the observed lifetimes and censoring times are independent. Let *F* and *C* be the sets of individuals for which $x_{i}$ is the lifetime or censoring, respectively. The total log-likelihood function for $\tau ={\left(\theta ,\beta_{1}^{T},\beta_{2}^{T}\right)}^{T}$ reduces to

(20) $$\begin{array}{ccc} l(\tau ) & = & r\log\left(\theta^{2}\;c_{\theta }\right)+\sum_{i\in F}\log\left(\frac{k_{i}}{\lambda_{i}^{k_{i}}}\right)+\sum_{i\in F}\left(k_{i}-1\right)\log\left(x_{i}\right)-\sum_{i\in F}{\left(\frac{x_{i}}{\lambda_{i}}\right)}^{k_{i}}+\\ & & \sum_{i\in F}\log\left\{2-\exp\left[-{\left(\frac{x_{i}}{\lambda_{i}}\right)}^{k_{i}}\right]\right\}-\sum_{i\in F}q\left(x_{i}|v_{i}\right)+\\ & & \sum_{i\in C}\log\left\{1-c_{\theta }\left\{1+\theta -\left[1+\theta +q\left(x_{i}|v_{i}\right)\right]\exp\left[-q\left(x_{i}|v_{i}\right)\right]\right\}\right\}, \end{array}$$

where *r* is the number of uncensored observations (failures) and $q\left(x_{i}|v_{i}\right)=\theta \left\{1-\exp\left[-{\left(\frac{x_{i}}{\lambda_{i}}\right)}^{k_{i}}\right]\right\}$. By maximizing the log-likelihood (20), the MLE of the vector of unknown parameters can be calculated. We use the R software to determine $\hat{\tau }$.

### 4.1. Residual Analysis

For the TLW regression model with censored observations, we present two types of residuals to evaluate deviations from the error assumptions and detect outliers. The deviance residuals have been used more frequently in the literature because they take into account the information of censored times. The TLW regression model can also use these residuals. A reliable method for detecting atypical observations and confirming that the fitted model is adequate is to plot the deviance residual against the observed times. It is possible to express the deviance residual as

(21) $$\begin{array}{c} r_{D_{i}}=sign\left(r_{M_{i}}\right){\left\{-2\left[r_{M_{i}}+\delta_{i}\log\left(\delta_{i}-r_{M_{i}}\right)\right]\right\}}^{1/2}, \end{array}$$

where

$$\begin{array}{c} r_{M_{i}}=\left\{\begin{array}{cc} 1+\log\left\{1-c_{\hat{\theta }}\left\{1+\hat{\theta }-\left[1+\hat{\theta }+\hat{q}\left(x_{i}|v_{i}\right)\right]\exp\left[-\hat{q}\left(x_{i}|v_{i}\right)\right]\right\}\right\} & if\;\delta_{i}=1,\\ \log\left\{1-c_{\hat{\theta }}\left\{1+\hat{\theta }-\left[1+\hat{\theta }+\hat{q}\left(x_{i}|v_{i}\right)\right]\exp\left[-\hat{q}\left(x_{i}|v_{i}\right)\right]\right\}\right\} & if\;\delta_{i}=0, \end{array}\right. \end{array}$$

is the martingale residual, $\delta_{i}=1$ means that the observation is uncensored, $\delta_{i}=0$ means that the observation is censored and

$$\hat{q}\left(x_{i}|v_{i}\right)=\hat{\theta }\left\{1-\exp\left[-{\left(\frac{x_{i}}{{\hat{\lambda }}_{i}}\right)}^{{\hat{k}}_{i}}\right]\right\}.$$

### 4.2. Simulation Study

To verify the accuracy of the MLEs of the TLW regression model, we carried out a simulation study for different censoring percentages and sample sizes n=100, 300, and 500. For each sample size, we carried out *N* = 1000 replicates and considered the approximate censoring percentages: 0%, 10% and 30%. A covariate $v_{1}∼$ binomial (1,0.5) is included from the following systematic components:

$$\lambda_{i}=\exp\left(\beta_{10}+\beta_{11}v_{1i}\right),\;\mathrm{and}\;k_{i}=\exp\left(\beta_{20}+\beta_{21}v_{1i}\right),$$

The inverse transformation method is used to obtain the lifetimes $x_{1},\cdots ,x_{n}$ from the TLW$\left(\lambda_{i},k_{i},\theta \right)$ distribution, and the censoring times $c_{1},\cdots ,c_{n}$ are determined from a uniform distribution (0,γ), where γ controls the censoring percentages. The true values used for generation are $\beta_{10}=0.3$, $\beta_{11}=0.4$, $\beta_{20}=0.2$, $\beta_{21}=0.5$, and θ=0.6.

The Results are checked for $\tau^{⊤}=\left({\hat{\beta }}_{10},{\hat{\beta }}_{11},{\hat{\beta }}_{20},{\hat{\beta }}_{21},\hat{\theta }\right)$ from MABs, MSEs, and AEs given in (18), where here Θ=τ. The simulation process is given by:

(i) Generate $v_{1i}∼$ binomial (n,1,0.5);

(ii) Calculate $\lambda_{i}=\exp\left(\beta_{10}+\beta_{11}v_{1i}\right)$ and $k_{i}=\exp\left(\beta_{20}+\beta_{21}v_{1i}\right)$;

(iii) Generate $x_{i}^{*}∼$ TLW $\left(n,\lambda_{i},k_{i},\theta \right)$;

(iv) Generate $c_{i}∼\mathrm{uniform}\left(0,\gamma \right)$;

(v) Calculate the survival times $x_{i}=\min\left(x_{i}^{*},c_{i}\right)$;

(vi) If $x_{i}^{*}<c_{i}$, then $\delta_{i}=1$; otherwise, $\delta_{i}=0$, for i=1,…,n, where δ is the censoring indicator.

(vii) Calculate AEs, biases, and MSEs.

Table 6 displays these values. It is verified that for all scenarios the averages of the estimates approach the true values of the parameters and the MABs and MSEs decrease as the sample size increases. These results illustrate that the estimates are consistent, even at higher censoring percentages.

## 5. Data Analysis

In order to demonstrate the superiority of the new distribution over some other models, we use two real datasets originating from different fields. We compare the fits of the TLW model to those of the parent Weibull model (W), the Kumarswamy–Weibull model (KW) from Cordeiro and Castro (2011) [4], the Weibull–Weibull model (WW) from Alzaatreh et al. [18], the Geometric–Poisson–Weibull model (GPW) from Nadarajah et al. (2013) [19], the Poisson–Weibull model (PW) from Ristic and Nadarajah (2013) [5] the beta-Weibull model (BW) from Eugene et al. (2002) [1], the Marshall–Olkin–Weibull model (MOW) from Marshall and Olkin (1997) [20] and the exponentiated generalized Weibull model (EGW) from Cordeiro et al. (2013) [21]. The cdfs of these models are provided in Appendix B. The parameter estimates are computed by maximizing (17) using the BFGS method available in *the adequacy model* package in the R software [22].

The considered models are compared according to a collection of statistics (AIC, CAIC, BIC, HQIC, minus maximum log-likelihood function (−ℓ)) which assess the relative degree of fit of these models to a dataset.

We also performed an application of the TLW regression model considering censored data. We compared different systematic components for the proposed new regression model and the Weibull regression model. In this part we use the RS algorithm in the *gamlss* package in the R software to maximize the log-likelihood function (20) and we use the AIC and global deviance (GD) statistics to select the most suitable models.

Dataset I: Temperature Dataset

This dataset, reported by Barakat et al. (2014) [23], depicts the average July temperatures (${}^{{}^{\circ}}$C) for Neuenburg, Switzerland, between 1864 and 1993. The observations are as follows.
19.020.118.417.419.721.021.419.219.920.420.917.220.217.818.115.619.421.716.216.419.020.619.020.715.817.716.817.118.118.418.718.718.419.218.018.720.719.419.217.422.021.419.316.818.216.215.922.117.515.316.517.417.018.318.315.318.221.517.021.618.218.117.618.222.619.917.117.217.319.420.120.117.019.417.516.817.019.918.219.218.520.819.521.115.821.321.218.822.318.616.818.217.218.418.721.116.317.418.019.521.216.817.420.718.419.818.720.518.318.218.219.220.218.217.419.216.317.420.323.419.220.219.319.018.820.319.720.719.618.1

The MLEs and 95% CIs for the model parameters are shown in Table 7. Table 8 provides the competence of the considered models.

**Figure 3:**
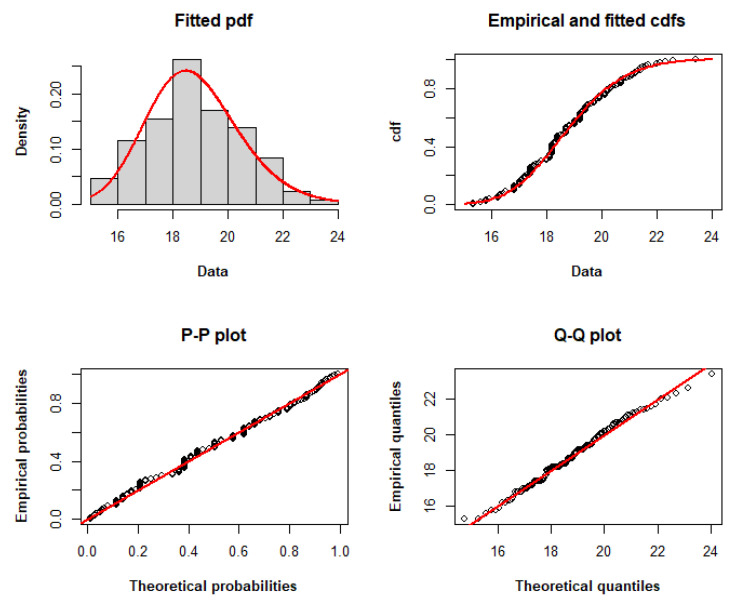
Histogram and fitted pdf, empirical and fitted cdfs, and P–P and Q–Q plots of the TLW model fitted to dataset I.

The TLW model fits the dataset with the lowest AIC, CAIC, BIC, HQIC, and minus log-likelihood among the other models, as determined by the adequacy statistics presented in Table 8. Therefore, it may be a viable option for modeling these data. Figure 3 compares the empirical and fitted distributions of the data, displaying the histogram and fitted pdf, the fitted and empirical cdfs, the P–P plot, and the Q–Q plot, respectively, to graphically explain the appropriateness of the TLW for modeling these data.

Dataset II: Breaking Stress of Carbon Fibers

The breaking stress of 64 single carbon fibers of gauge length 10 mm (Cheng and Traylor (1970) [24]). The observations are as follows.
1.9012.1322.2032.2282.2572.352.3612.3962.3972.44502.4542.4542.4742.5182.5222.5252.5322.5752.6142.6162.6182.6242.6592.6752.7382.742.8562.9172.9282.9372.9372.9772.9963.033.1253.1393.1453.223.2233.2353.2433.2643.2723.2943.3323.3463.3773.4083.4353.4933.5013.5373.5543.5623.6283.8523.8713.8863.9714.0244.0274.2254.3955.02



**Figure 4:**
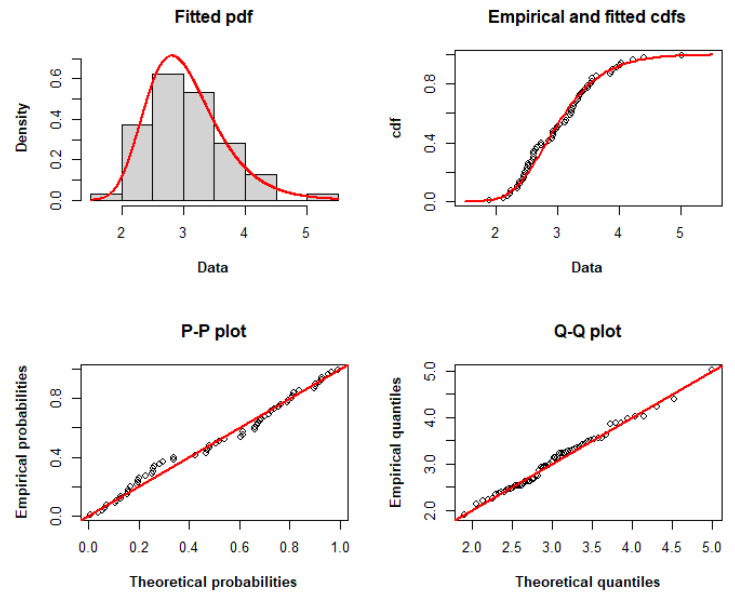
Histogram and fitted pdf, empirical and fitted cdfs, and P–P and Q–Q plots of the TLW model fitted to dataset II.

Table 9 displays the MLEs and 95% CIs for the model parameters, demonstrating the validity of the considered models. According to Table 10, the TLW model fits the dataset with the lowest AIC, CAIC, BIC, HQIC, and minus log-likelihood among the other models. Therefore, it may be a viable option for modeling these data. Figure 4 compares the empirical and fitted distributions of the data, displaying the histogram and fitted pdf, the fitted and empirical cdfs, the P–P plot and the Q–Q plot to graphically demonstrate the appropriateness of the TLW for modeling these data.

Dataset III: COVID-19

In this application we consider the regression model for censored data. This dataset refers to patients hospitalized with COVID-19. The disease is caused by the pathogen identified as a new coronavirus, denominated severe acute respiratory syndrome coronavirus-2 (SARS-CoV-2). The epidemiological data were tallied by the Health Information System of the Brazilian government, and are available at https://opendatasus.saude.gov.br/dataset/srag-2020 (accessed on 1 May 2023).

This study involved 195 patients hospitalized in the city of Campinas, state of São Paulo, in May 2020, with infection confirmed by RT-PCR and classified as SARS caused by COVID-19. The survival time consisted of the time in days from the date of first symptoms to the date of evolution of the case, either death (failure) or end of observation (censoring). The censoring percentage was 56.92% and the following variables were considered: (i=1,⋯,195):

- $x_{i}$: observed time (in days);
- ${\mathrm{cens}}_{i}$: censoring indicator (0= censored, 1= observed lifetime);
- $v_{i1}$: sex (1= male, 0= female);
- $v_{i2}$: age (in years).

**Figure 5:**
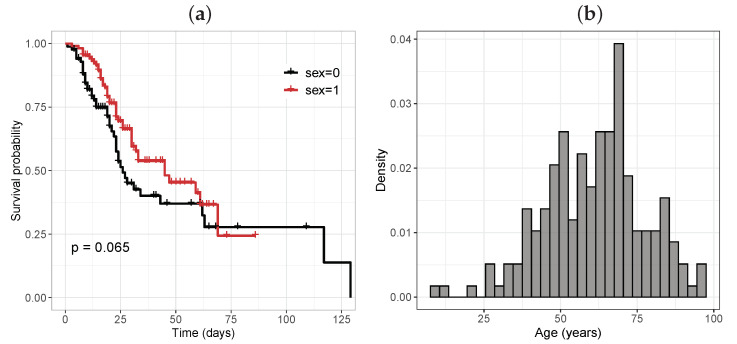
(**a**) Kaplan–Meier survival curve for the sex variable (1= male, 0= female); (**b**) histogram of the age variable.

There were 110 male patients (56.41%), of whom 42 (38.18%) died, while of the 85 women (43.58%), there were 42 deaths (49.41%). Figure 5a presents the Kaplan–Meier survival curve broken down by sex. It can be seen that men had a higher risk of death. Figure 5b depicts the histogram of the ages, where the greatest frequency was in the category from 50 to 75 years old.

We compared the TLW regression model with the Weibull regression model based on the following systematic components:

$$\mathrm{Systematic}=\left\{\begin{array}{c} M_{0}:\log\left(\lambda_{i}\right)=\beta_{10}\;\mathrm{and}\;\log\left(k_{i}\right)=\beta_{20};\\ M_{1}:\log\left(\lambda_{i}\right)=\beta_{10}+\beta_{11}v_{i1}+\beta_{12}v_{i2}\;\mathrm{and}\;\log\left(k_{i}\right)=\beta_{20};\\ M_{2}:\log\left(\lambda_{i}\right)=\beta_{10}\;\mathrm{and}\;\log\left(k_{i}\right)=\beta_{20}+\beta_{21}v_{i1}+\beta_{22}v_{i2};\\ M_{3}:\log\left(\lambda_{i}\right)=\beta_{10}+\beta_{11}v_{i1}+\beta_{12}v_{i2}\;\mathrm{and}\;\log\left(k_{i}\right)=\beta_{20}+\beta_{21}v_{i1}+\beta_{22}v_{i2}. \end{array}\right.$$

**Figure 6:**
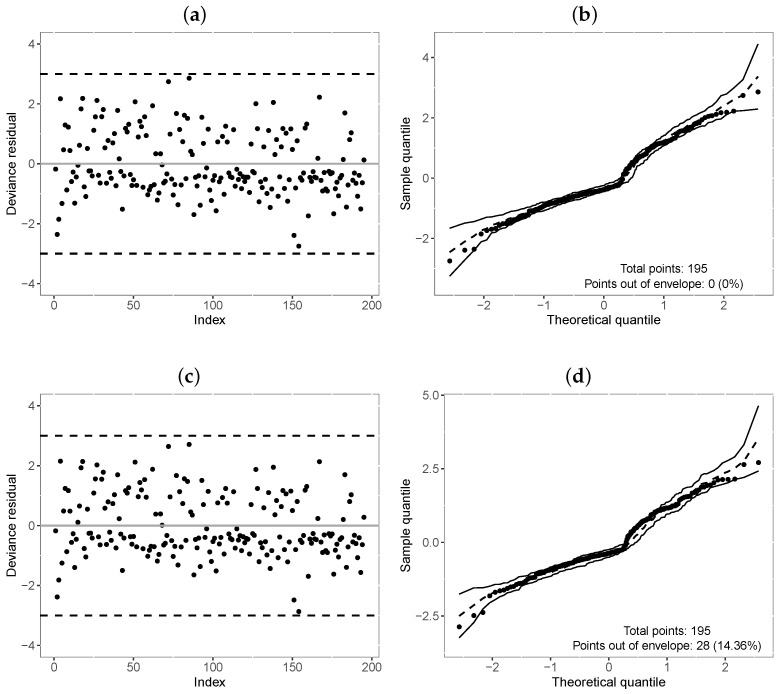
Index plot and normal probability plot with envelope of the deviance residual from the fitted regressions model to the COVID-19 data. (**a**,**b**): $M_{3}$-TLW; (**c**,**d**): $M_{3}$-Weibull.

**Figure 7:**
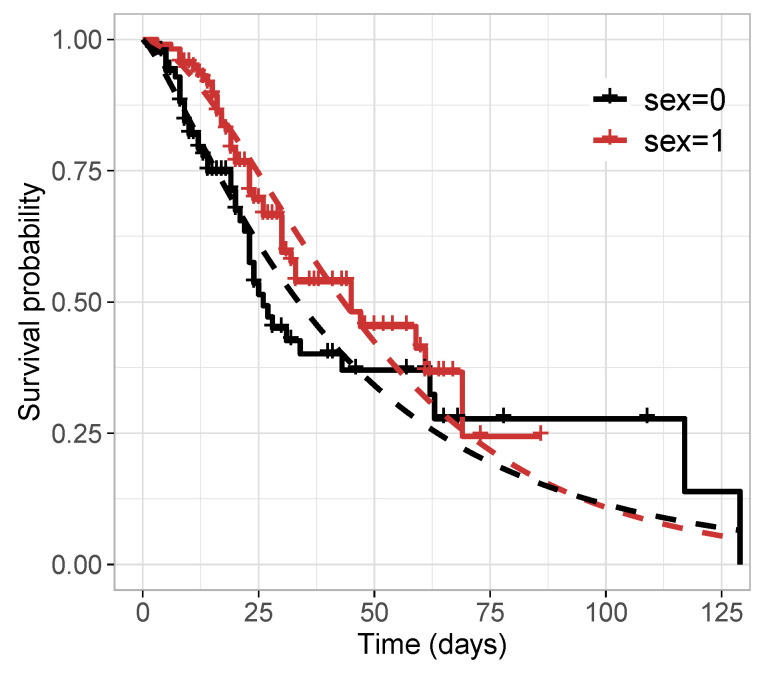
Kaplan–Meier survival curve and estimated survival functions from the $M_{3}$-TLW by sex.

Table 11 reports the values of the selection criteria of the models, in which the $M_{3}$-TLW model was superior to the others. We also compared this model with the $M_{3}$-Weibull model by means of the residuals in Figure 6. In turn, Figure 6a,c illustrate the residuals versus the index of the observations, showing that both models have residuals with random behavior around zero, and no point is outside the interval (−3,3). Nevertheless, Figure 6b,d indicate that the TLW model behaved better, with all the points within the simulated envelope, denoting its superiority. Finally, we illustrate the Kaplan–Meier curves and estimated survival curves in Figure 7 for the TLW model, showing that this model is able to capture the non-proportional curves of this dataset. The results of this model are shown in Table 12. Some conclusions can be obtained as follows.

Interpretations for λ:

- A significant difference exists between men and women in relation to survival time (men have shorter survival). Various other studies have also indicated significant differences between the sexes (see [25,26]);
- The survival time declines with advancing age. This result corroborates the findings of several studies that have indicated that older age is a predictor of higher mortality caused by COVID-19 (see [27,28,29]).

Interpretations for *k*:

- A significant difference exists between men and women with regard to the variability in the survival time;
- In relation to age, the variability in survival time increased with older age of the patients.

Dataset IV: Post-harvested

**Figure 8:**
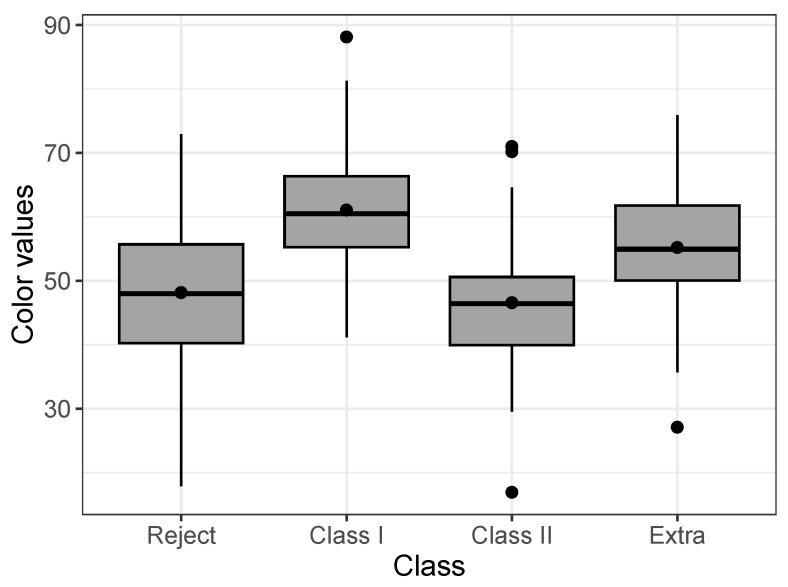
Boxplot of colors by class for the Post-harvested dataset.

In this application, we consider the regression model for uncensored data. These data refer to Musa acuminata banana species from a banana plantation in the Philippines. A total of n=194 banana tiers were chosen randomly, in which the numerical values of the RGB colors (red, green, and blue) were obtained from images taken by hardware of four banana classes, extra class, class I, class II, and reject, where the classes contain 65, 49, 30, and 50 samples, respectively. The dataset is available in the repository: https://data.mendeley.com/datasets/zk3tkxndjw/2 (accessed on 20 May 2023) and more details can be seen in [30]. Each banana tier sample was captured with a white background in six different views: front, back, left, right, top, and bottom views. Here, we consider the values of B in front view. Figure 8 displays a boxplot by class, it is possible to observe differences between the colors according to the class.

The variables considered are (i=1,…,194):

- $x_{i}$: color value;
- $v_{ij}$: banana class (factor with four levels, defined by three variable dummies j=1,2,3).

We verified the relationship between colors and classes from the TLW and Weibull models according to the following systematic components:

$$\mathrm{Systematic}=\left\{\begin{array}{c} M_{0}:\log\left(\lambda_{i}\right)=\beta_{10}\;\mathrm{and}\;\log\left(k_{i}\right)=\beta_{20};\\ M_{1}:\log\left(\lambda_{i}\right)=\beta_{10}+\beta_{1j}v_{ij}\;\mathrm{and}\;\log\left(k_{i}\right)=\beta_{20};\\ M_{2}:\log\left(\lambda_{i}\right)=\beta_{10}\;\mathrm{and}\;\log\left(k_{i}\right)=\beta_{20}+\beta_{2j}v_{ij};\\ M_{3}:\log\left(\lambda_{i}\right)=\beta_{10}+\beta_{1j}v_{ij}\;\mathrm{and}\;\log\left(k_{i}\right)=\beta_{20}+\beta_{2j}v_{ij}. \end{array}\right.$$

**Figure 9:**
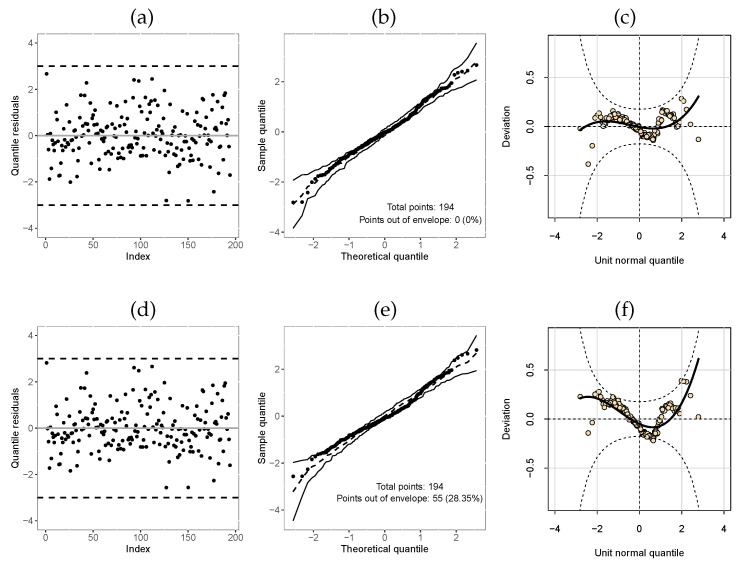
Index plot, normal probability plot with envelope, and worm plot of the quantile residuals from the regression models fitted to the Post-harvested dataset: (**a**–**c**): $M_{3}$-TLW; (**d**–**f**): $M_{3}$-Weibull.

Table 13 displays the AIC and GD values for these fitted models, in which it can be seen that the $M_{3}$-TLW model obtained the lowest values, being able to be chosen as the best model. In addition, we compare the $M_{3}$-TLW and the $M_{3}$-Weibull from the quantile residues (Figure 9). These plots agree with the results of Table 13, there is a high percentage of points outside the confidence band of the Weibull model (Figure 9e) and many deviations also from the confidence band worm plot confidence (Figure 9f).

Finally, Table 14 presents MLEs, SEs, and *p*-values of the model $M_{3}$-TLW, in which classes I, II, and extra are compared with the rejected class. We can obtain the following conclusions: there is a significant difference between the color of class 1 and the rejects. Its effect is positive, that is, it presented higher color values. Class II and the extra class do not present a significant difference with the rejected class. The extra class and class I’s colors affect the shape of the distribution compared to the reject class’s color.

## 6. Conclusions

In this study, we propose a new class of distributions called the truncated Lindley-*G* (TLG) distribution with application to the truncated Lindley–Weibull (TLW) distribution with three parameters. Several structural properties of the TLG distribution, including an expansion of the density function, critical points, explicit expressions of the ordinary and incomplete moments, mean deviation, generating function, entropy, and quantile function, are discussed. The parameters of the model are estimated using the maximum likelihood technique. We fitted the TLW model to two sets of data to demonstrate the effectiveness of the proposed distribution. In comparison to the Kumarswamy–Weibull, Weibull–Weibull, Geometric–Poisson–Weibull, Poisson–Weibull, beta-Weibull, Marshall–Olkin–Weibull, and exponentiated generalized Weibull distributions, the proposed model had a better fit on four datasets. However, the goodness-of-fit measures for our model were not drastically better than the comparison models that are currently used in statistical analyses. Based on this new distribution, we propose a TLW regression model with two systematic components very suitable for modeling censored and uncensored data. Several simulation studies are performed for different parameter settings, sample sizes, and censoring percentages. We anticipate the further application of the proposed model in disciplines such as engineering, survival and lifetime data, and economics.

## Figures and Tables

**Table 1:** Previous work on TG models.

Model	Author(s)	cdf
Poisson-*G*	Ristic and Nadarajah (2013) [5]	$\frac{1-e^{-a\;G^{b}\left(x\right)}}{1-e^{-a}}$
Truncated-exponential skew-symmetric-*G*	Nadarajah et al. (2014a) [6]	$\frac{1-e^{-aG(x)}}{1-e^{-a}}$
Truncated-Fréchet-*G*	Abid and Abdulrazak (2017) [7]	$e^{a\left[1-G{\left(x\right)}^{-b}\right]}$
Truncated inverted Kumaraswamy-*G*	Bantan et al. (2019) [8]	$\frac{{\left[1-{\left(1+G(x)\right)}^{-a}\right]}^{b}}{{\left(1-2^{-a}\right)}^{b}}$
Type II truncated Fréchet-*G* (truncated inverse Weibull-*G*)	Aldahlan et al. (2019) [9]	$1-e^{1-{\left(1-G(x)\right)}^{-a}}$
Exponentiated truncated inverse Weibull-*G*	Almarashi et al. (2020) [10]	${\left[1-e^{1-{\left(1-G(x)\right)}^{-a}}\right]}^{b}$
Truncated Burr-*G*	Jamal et al. (2020) [11]	$\frac{1-{\left[1+G^{c}\left(x\right)\right]}^{-k}}{1-2^{-k}}$
Truncated Muth-*G*	Almarashi et al. (2021) [12]	$\frac{1-e^{\left[\alpha \;G\left(x\right)-\left(e^{\alpha G(x)}-1\right)/\alpha \right]}}{1-e^{\left[\alpha -\left(e^{\alpha }-1\right)/\alpha \right]}}$
Truncated generalized Fréchet-*G*	ZeinEldin et al. (2021) [13]	$\frac{1-{\left[1-e^{-\alpha /G(x)}\right]}^{b}}{1-{\left(1-e^{-\alpha }\right)}^{b}}$
Truncated inverse Lomax-*G*	Algarni et al. (2021) [14]	$1-2^{\alpha }{\left[1+{\left(1-G\left(x\right)\right)}^{-1}\right]}^{-\alpha }$
Truncated Burr X-*G*	Bantan et al. (2021) [15]	$\frac{{\left(1-e^{-\alpha^{2}G^{2}\left(x\right)}\right)}^{\theta }}{{\left(1-e^{-\alpha^{2}}\right)}^{\theta }}$

**Table 2:** Number of roots of equation $A\left(z_{0}\right)=B_{\theta ,k}\left(z_{0}\right)$ in (16) when varying the parameters θ and $\tau^{*}$.

	$\tau^{*}$	≤$-\frac{1}{e}$	$>-\frac{1}{e}$∧<0	≥0
θ				
>0		single root	single root	no root
<0		no root	two roots	single root

**Table 3:** Shapes of TLW pdf when varying the parameters θ and $\tau^{*}$.

	$\tau^{*}$	≤$-\frac{1}{e}$	$>-\frac{1}{e}$∧<0	≥0
θ				
>0		Unimodality	Unimodality	Decreasing
<0		Decreasing	Decreasing–increasing–decreasing	Unimodality

**Table 4:** Average estimates from simulations of the TLW distribution.

Parameters				ME		
θ	k	λ	n	$\hat{\theta }$	$\hat{k}$	$\hat{\lambda }$
0.5	0.5	0.5	20	0.3927	0.5982	0.5915
			50	0.3959	0.5104	0.5143
			100	0.5818	0.5086	0.4582
			150	0.5782	0.5078	0.4737
			300	0.5052	0.5003	0.5012
0.5	2	2	20	0.3821	2.1788	2.4017
			50	0.3828	2.1724	2.3466
			100	0.5195	2.1554	2.1472
			150	0.4945	2.1159	2.0617
			300	0.4984	2.0195	2.0324
2	2	0.5	20	2.8780	2.8245	0.6463
			50	2.6245	2.2245	0.4403
			100	2.1419	2.0419	0.5388
			150	2.0545	1.9545	0.5036
			300	2.0044	1.9994	0.5004
3	0.5	3	20	2.4702	0.6231	2.2937
			50	2.6202	0.3798	3.2610
			100	2.8369	0.4631	3.2424
			150	2.8535	0.5146	3.1024
			300	2.9823	0.5018	3.0635
2	5	2	20	2.7795	5.9724	1.6501
			50	2.3405	5.4046	2.2949
			100	1.8551	5.1855	1.7808
			150	2.0733	5.0733	2.0985
			300	1.9930	4.9790	2.0104
5	3	3	20	6.1274	3.2987	2.6354
			50	5.2781	3.1288	2.6674
			100	4.8956	3.1146	2.8674
			150	4.9895	3.0985	2.9631
			300	5.0013	3.0043	2.9985
5	4	2	20	4.4533	4.5847	2.8655
			50	5.2474	3.8812	2.4652
			100	4.9521	3.8932	2.4245
			150	5.1124	3.9958	2.1135
			300	4.9821	4.0024	2.0075

**Table 5:** MABs and MSEs from simulations of the TLW distribution.

Parameters				MAB			MSE		
θ	k	λ	n	$\hat{\theta }$	$\hat{k}$	$\hat{\lambda }$	$\hat{\theta }$	$\hat{k}$	$\hat{\lambda }$
0.5	0.5	0.5	20	0.1073	0.0982	0.0915	0.4927	0.3251	0.2520
			50	0.1041	0.0104	0.0143	0.1538	0.1607	0.2497
			100	0.0818	0.0086	0.0418	0.1353	0.0656	0.1960
			150	0.0782	0.0078	0.0263	0.0230	0.0421	0.0540
			300	0.0052	0.0003	0.0012	0.0110	0.0215	0.0301
0.5	2	2	20	0.1179	0.1788	0.4018	0.3210	0.4573	0.4200
			50	0.1172	0.1724	0.3466	0.1420	0.2923	0.2584
			100	0.0195	0.1554	0.1472	0.0732	0.0832	0.1453
			150	0.0055	0.1159	0.0617	0.0612	0.0549	0.1087
			300	0.0016	0.0195	0.0324	0.0139	0.0490	0.0359
2	2	0.5	20	0.8780	0.8245	0.1463	0.7810	0.5427	0.7147
			50	0.6245	0.2245	0.0597	0.6531	0.4417	0.6984
			100	0.1419	0.0490	0.0388	0.1456	0.2542	0.1825
			150	0.0545	0.0455	0.0036	0.0574	0.0088	0.0821
			300	0.0044	0.0006	0.0004	0.0035	0.0015	0.0674
3	0.5	3	20	0.5298	0.1231	0.7063	1.0745	0.8945	0.7984
			50	0.3798	0.1202	0.2610	0.6870	0.3017	0.5203
			100	0.1631	0.0369	0.2424	0.2153	0.1465	0.2257
			150	0.1465	0.0146	0.1024	0.1040	0.0896	0.0357
			300	0.0177	0.0018	0.0635	0.0862	0.0651	0.0089
2	5	2	20	0.7795	0.9724	0.3499	0.8691	1.2143	1.1401
			50	0.3405	0.4046	0.2949	0.4041	0.9674	0.5189
			100	0.1449	0.1855	0.2192	0.3540	0.6307	0.5021
			150	0.0733	0.0733	0.0985	0.0957	0.0390	0.1008
			300	0.0070	0.0210	0.0104	0.0068	0.0107	0.0096
5	3	3	20	1.1274	0.2987	0.3646	1.8752	2.0145	1.4571
			50	0.2781	0.1288	0.3326	1.0587	1.5124	0.6501
			100	0.1044	0.1146	0.1326	0.6321	0.8210	0.0893
			150	0.0105	0.0985	0.0369	0.2480	0.6347	0.0101
			300	0.0013	0.0043	0.0015	0.0472	0.0086	0.0054
5	4	2	20	0.5467	0.5847	0.8655	2.1768	1.7456	1.9087
			50	0.2474	0.1188	0.4652	0.8740	1.0157	0.9889
			100	0.0479	0.1068	0.4245	0.6531	0.8751	0.2350
			150	0.1124	0.0042	0.1135	0.0478	0.1450	0.0842
			300	0.0179	0.0024	0.0075	0.0023	0.0541	0.0357

**Table 6:** Simulation results of TLW regression models for different censoring percentages (%) with true values: $\beta_{10}=0.3$, $\beta_{11}=0.4$, $\beta_{20}=0.2$, $\beta_{21}=0.5$, and θ=0.6.

		n=100				n=300				n=500		
%	θ	**AEs**	**MABs**	**MSEs**		**AEs**	**MABs**	**MSEs**		**AEs**	**MABs**	**MSEs**
0%	$\beta_{10}$	0.2949	−0.0051	0.0239		0.3018	0.0018	0.0091		0.3056	0.0056	0.0054
	$\beta_{11}$	0.4077	0.0077	0.0232		0.3994	−0.0006	0.0071		0.3960	−0.0040	0.0044
	$\beta_{20}$	0.2234	0.0234	0.0137		0.2073	0.0073	0.0046		0.2087	0.0087	0.0026
	$\beta_{21}$	0.5014	0.0014	0.0260		0.5019	0.0019	0.0086		0.4971	−0.0029	0.0049
	θ	0.6323	0.0323	0.2064		0.6241	0.0241	0.0984		0.6286	0.0286	0.0569
10%	$\beta_{10}$	0.2933	−0.0067	0.0219		0.3012	0.0012	0.0090		0.3018	0.0018	0.0050
	$\beta_{11}$	0.4062	0.0062	0.0228		0.3990	−0.0010	0.0075		0.3991	−0.0009	0.0041
	$\beta_{20}$	0.2192	0.0192	0.0138		0.2100	0.0100	0.0051		0.2054	0.0054	0.0030
	$\beta_{21}$	0.5064	0.0064	0.0283		0.4983	−0.0017	0.0089		0.5028	0.0028	0.0054
	θ	0.6188	0.0188	0.1765		0.6225	0.0225	0.0908		0.6144	0.0144	0.0491
30%	$\beta_{10}$	0.2902	−0.0098	0.0253		0.2997	−0.0003	0.0101		0.3033	0.0033	0.0057
	$\beta_{11}$	0.4114	0.0114	0.0266		0.3987	−0.0013	0.0088		0.3969	−0.0031	0.0055
	$\beta_{20}$	0.2313	0.0313	0.0208		0.2093	0.0093	0.0060		0.2072	0.0072	0.0033
	$\beta_{21}$	0.5005	0.0005	0.0404		0.5013	0.0013	0.0111		0.4980	−0.0020	0.0065
	θ	0.6306	0.0306	0.1611		0.6125	0.0125	0.0960		0.6138	0.0138	0.0518

**Table 7:** Estimates of TLW parameters for dataset I.

	MLE	Std. Err	Inf. 95% CI	Sup. 95% CI
θ	−28.44948	28.085548	−32.53708	−25.44727
*k*	3.454494	0.9554519	1.581842	5.327145
λ	12.75564	2.3338198	8.181439	17.32985

**Table 8:** Competence of the models for the dataset.

Distribution	No. of Estimated Parameters	AIC	CAIC	BIC	HQIC	−ℓ
TLW	3	507.124	507.314	515.726	510.619	250.562
W	2	524.667	524.762	530.402	526.998	260.334
KW	4	510.612	510.932	522.082	515.273	251.306
WW	4	528.667	528.987	540.137	533.328	260.334
GPW	4	512.800	513.120	524.270	517.460	252.400
PW	4	513.232	513.552	524.702	517.893	252.616
BW	4	511.523	511.843	522.993	516.184	251.762
MOW	3	513.522	513.713	522.125	517.018	253.761
EGW	4	512.706	513.026	524.176	517.367	252.353

**Table 9:** Estimates of TLW parameters for dataset II.

	MLE	Std. Err	Inf. 95% CI	Sup. 95% CI
θ	−49.54747	3.8976505	−57.18672	−43.9367
*k*	1.374038	0.4973775	0.399197	2.348880
λ	1.029519	0.6568050	−0.257795	2.316833

**Table 10:** Competence of the models for dataset II.

Distribution	No. of Estimated Parameters	AIC	CAIC	BIC	HQIC	−ℓ
TLW	3	118.197	118.597	124.673	120.748	56.098
W	2	129.933	130.130	134.251	131.634	62.967
KW	4	121.642	122.320	130.278	125.044	56.821
WW	4	133.933	134.611	142.569	137.335	62.967
GPW	4	122.118	122.796	130.754	125.520	57.059
PW	4	123.742	124.420	132.377	127.144	57.871
BW	4	121.285	121.963	129.921	124.687	56.643
MOW	3	122.570	122.970	129.047	125.122	58.285
EGW	4	121.883	122.561	130.519	125.285	56.942

**Table 11:** AIC and GD values for TLW and Weibull regression models with different structures for COVID-19 data.

Model	TLW					Weibull			
	$M_{0}$	$M_{1}$	$M_{2}$	$M_{3}$		$M_{0}$	$M_{1}$	$M_{2}$	$M_{3}$
AIC	854.947	821.162	828.469	814.707		855.651	823.412	848.348	817.815
GD	848.947	811.162	818.469	800.707		851.651	815.412	840.348	805.815

**Table 12:** MLEs, SEs, and *p*-values for the $M_{3}$-TLW regression fitted to COVID-19 data.

	MLEs	SEs	*p*-Values
$\beta_{10}$	7.8325	0.2995	<0.01
$\beta_{11}$	−0.419	0.1432	<0.01
$\beta_{12}$	−0.0467	0.0040	<0.01
$\beta_{20}$	−0.4240	0.1690	0.01
$\beta_{21}$	0.4605	0.0939	<0.01
$\beta_{22}$	0.0096	0.0027	<0.01
θ	3.6408	0.3289	<0.01

**Table 13:** AIC and GD values for TLW and Weibull regression models with different structures for the Post-harvested dataset.

Model	TLW					Weibull			
	$M_{0}$	$M_{1}$	$M_{2}$	$M_{3}$		$M_{0}$	$M_{1}$	$M_{2}$	$M_{3}$
AIC	1520.226	1491.587	1487.429	1482.161		1519.985	1495.154	1514.562	1486.809
GD	1514.226	1479.587	1475.429	1464.161		1515.985	1485.154	1504.562	1470.809

**Table 14:** MLEs, SEs, and *p*-values for the $M_{3}$-TLW regression fitted to the Post-harvested dataset.

	MLEs	SEs	*p*-Values
$\beta_{10}$	4.1417	0.0380	<0.01
$\beta_{11}$	0.1469	0.0471	<0.01
$\beta_{12}$	−0.0800	0.0482	0.0987
$\beta_{13}$	0.0354	0.0424	0.4044
$\beta_{20}$	1.5394	0.1087	<0.01
$\beta_{21}$	0.4443	0.1825	0.0159
$\beta_{22}$	0.1994	0.1532	0.1949
$\beta_{23}$	0.5335	0.1407	<0.01
θ	3.2938	0.2847	

## Data Availability

Stated in the text.

## Appendix A

The UTL model has the following properties:

(1) Moments
  The UTL distribution’s *k*th raw moment (k=1,2,⋯) is given by
  $$\mu_{k}^{\prime }=\theta^{2}\;C_{\theta }\;\left[-\frac{e^{-\theta }}{\theta }+\left(1+\frac{k+1}{\theta }\right)\;d_{k}\right],$$
  where $d_{k}=\int_{0}^{1}x^{k}e^{-\theta \;x}dx$. Using integration by parts, $d_{k}$ can be calculated recursively by
  $$d_{k}=\frac{1}{\theta }\left(k\;d_{k-1}-e^{-\theta }\right),\;\;\;k=1,2,\cdots ,$$
  and
  $$d_{0}=\frac{1}{\theta }\left(1-e^{-\theta }\right).$$
  The first three moments are
  $$\begin{array}{ccc} \mu_{1}^{\prime } & = & \frac{\left(2+\theta -2e^{-\theta }-3\theta e^{-\theta }-2\theta^{2}e^{-\theta }\right)C_{\theta }}{\theta },\\ \mu_{2}^{\prime } & = & \frac{\left(6+2\;\theta -6e^{-\theta }-8\;\theta \;e^{-\theta }-5\;\theta^{2}e^{-\theta }-2\theta^{3}e^{-\theta }\right)C_{\theta }}{\theta^{2}},\;\mathrm{and}\\ \mu_{3}^{\prime } & = & \frac{\left(24+6\theta -24e^{-\theta }-30\theta e^{-\theta }-18\theta^{2}e^{-\theta }-7\theta^{3}e^{-\theta }-2\theta^{4}e^{-\theta }\right)C_{\theta }}{\theta^{3}}. \end{array}$$
  The *k*th incomplete moment of *X* is given by
  $$\begin{array}{ccc} I_{X}\left(t;k\right) & = & E\left(X^{k}|X\leq t\right)=\int_{0}^{t}x^{k}f_{UTL}\left(x\right)dx=\theta^{2}\;C_{\theta }\;\left[-\frac{t^{k}e^{-\theta t}}{\theta }+\left(1+\frac{k+1}{\theta }\right)\;d_{t,k}\right], \end{array}$$
  where $d_{t,k}=\int_{0}^{t}x^{k}e^{-\theta \;x}$. Using integration by parts, $d_{t,k}$ can be calculated recursively by
  $$d_{t,k}=\frac{1}{\theta }\left(k\;t^{k}\;d_{t,k-1}-e^{-\theta \;t}\right),\;\;\;k=1,2,\cdots ,$$
  and
  $$d_{t,0}=\frac{1}{\theta }\left(1-e^{-\theta \;t}\right).$$
(2) Mode
  The mode of the UTL distribution is
  $$\begin{array}{c} Mode=\left\{\begin{array}{cc} \frac{1-\theta }{\theta } & if\;0.5\leq \theta \leq 1,\\ 0 & if\;\theta >1,\\ 1 & if\;\theta <0.5. \end{array}\right. \end{array}$$
(3) Quantile Function
  Therefore The UTL distribution’s qf is
  $$Q_{UTL}\left(u\right)=-1-\frac{1}{\theta }-\frac{1}{\theta }W\left(\left(uC_{\theta }^{-1}-\theta -1\right)\;e^{-\theta -1}\right),\;\;\;\;u\in \left(0,1\right),$$
  where W(x) is the Lambert function satisfying $W\left(x\right)\;e^{W(x)}=x$ for x∈[−1/e,∞) (see Corless et al. [31] for the definition and properties of the Lambert function).
  Therefore, the median of the UTL distribution is simply $M=Q_{UTL}\left(0.5\right)$, that is,
  $$M=-1-\frac{1}{\theta }-\frac{1}{\theta }W\left(\left(0.5\;C_{\theta }^{-1}-\theta -1\right)\;e^{-\theta -1}\right).$$
(4) Mean Deviations
  The UTL distribution’s mean deviation about the mean μ=E(X) is given by
  $$\begin{array}{ccc} \delta_{1} & = & \int_{0}^{1}\left|x-\mu \right|f_{UTL}\left(x\right)dx\\ & = & \int_{0}^{\mu }\left(\mu -x\right)f_{UTL}\left(x\right)dx+\int_{\mu }^{1}\left(x-\mu \right)f_{UTL}\left(x\right)dx\\ & = & 2\mu \;F_{UTL}\left(\mu \right)-2\int_{0}^{\mu }x\;f_{UTL}\left(x\right)dx=2\left[\mu F_{UTL}\left(\mu \right)-\;I_{X}\left(\mu ;1\right)\right] \end{array}$$
  and the mean deviation about the median *M* is
  $$\delta_{2}=\int_{0}^{1}|x-M|\;f_{UTL}\left(x\right)\;dx=\mu -2\;I_{X}\left(M;1\right),$$
  where $I_{X}\left(t;k\right)$ is the *k*th incomplete moment.
(5) Moment Generating Function
  The UTL distribution’s moment generating function (mgf) can be expressed as
  $$M\left(t\right)=\int_{0}^{1}e^{tx}f_{UTL}\left(x\right)dx=\theta^{2}C_{\theta }\left[\frac{2t\;e^{-(\theta -t)}-2\theta \;e^{-(\theta -t)}-e^{-(\theta -t)}-t+\theta +1}{t^{2}-2\;\theta \;t+\theta^{2}}\right].$$

## Appendix B

- The cdf of the Kumaraswamy-*G* model is given by
  $$F\left(x\right)=1-{\left[1-G^{a}\left(x\right)\right]}^{b},\;\;\;\;a,b>0$$
- The cdf of the Weibull-*G* model is given by
  $$F\left(x\right)=1-\exp\left\{-{\left[-\frac{\log(1-G(x))}{b}\right]}^{a}\right\},\;\;\;\;a,b>0$$
- The cdf of the Geometric-Poisson-*G* model is given by
  $$F\left(x\right)=\frac{\exp[-a+aG(x)]-\exp(-a)}{1-\exp(-a)-b+b\exp[-a+aG(x)]},\;\;a>0,\;0<b<1$$
- The cdf of the Poisson-*G* model is given by
  $$F\left(x\right)=\frac{1-\exp\left[-a\;G^{b}\left(x\right)\right]}{1-\exp\left(-a\right)},\;\;\;a,b>0$$
- The cdf of the Beta-*G* model is given by
  $$F\left(x\right)=I_{G(x)}\left(a,b\right)$$
  where $I_{x}\left(a,b\right)=\int_{0}^{x}t^{a-1}{\left(1-t\right)}^{b-1}dt/B\left(a,b\right)$ is the regularized incomplete beta function, and $B\left(a,b\right)=\int_{0}^{1}t^{a-1}{\left(1-t\right)}^{b-1}dt$ is the beta function.
- The cdf of the Marshall–Olkin-*G* model is given by
  $$F\left(x\right)=\frac{G(x)}{a+\left(1-a\right)G\left(x\right)},\;\;\;\;\;a>0$$
- The cdf of the exponentiated generalized-*G* model is given by
  $$F\left(x\right)={\left[1-{\left[1-G\left(x\right)\right]}^{a}\right]}^{b},\;\;\;\;\;a,b>0$$
